# Integrating a Brief Behavioral Intervention Into Case Management for Mothers With Perinatal Substance Use Disorder: Nonrandomized Pilot Feasibility Study

**DOI:** 10.2196/75132

**Published:** 2025-12-30

**Authors:** Amritha Bhat, Mary C Curran, Mary Jane Lohr, Haley Smith, Brittany Blanchard, Susan A Stoner, Therese Grant, Nancy Grote

**Affiliations:** 1 Department of Psychiatry and Behavioral Sciences, University of Washington, 1959 NE Pacific Street, Box 356560, Seattle, WA, 98195, United States, 1 2065433117; 2 Department of Rehabilitation Medicine, University of Washington, Seattle, WA, United States; 3 School of Nursing, University of Washington, Seattle, WA, United States; 4 School of Social Work, University of Washington, Seattle, WA, United States

**Keywords:** perinatal, mental health, substance use, case management, feasibility

## Abstract

**Background:**

Perinatal substance use disorders (SUD) are frequently comorbid with depression, anxiety, and posttraumatic stress disorder (PTSD), contributing to adverse maternal and child outcomes. Access to integrated mental health support within existing SUD service frameworks is limited, particularly for pregnant and parenting individuals facing socioeconomic and psychosocial instability. Promoting Healthy Families (PHF) is a brief behavioral intervention designed for delivery by case managers serving high-risk perinatal populations with substance use within programs such as Parent Child Assistance Program (PCAP).

**Objective:**

This study aimed to evaluate the feasibility of integrating PHF into intensive case management for pregnant and postpartum clients with at-risk perinatal substance use, and to assess preliminary outcomes of measures of maternal depression, anxiety, and PTSD symptoms.

**Methods:**

In this nonrandomized pilot study (April 2018-September 2021), eligible clients were allocated to either PCAP alone (control) or to PHF delivered within PCAP (intervention). Case managers completed an anonymous feasibility survey addressing ease of delivery and fit with their workflow. Participating clients completed the Patient Health Questionnaire-9 (PHQ-9), Generalized Anxiety Disorder-7 Scale, and PTSD (posttraumatic stress disorder) Checklist (PCL-6) at baseline and at 4, 6, and 12 months. Data collection overlapped with the COVID-19 pandemic, which affected service access and delivery.

**Results:**

CMs and 1 program supervisor (n=10) reported that PHF was feasible to deliver within PCAP, and respondents indicated clients benefited somewhat (70%) or a lot (30%). Most (70%) noted an increase in workload and recommended additional supervision and training. The pilot study enrolled 58 clients (29 PHF+PCAP and 29 PCAP), with 60% (35/58) completing all follow-up assessments. While differences between groups over time were not statistically significant, changes were in the predicted direction for PHQ-9 and PCL-6 scores. Symptom improvement rates were high: In the PHF+PCAP group, 85% (25/29) showed ≥5-point decreases in PHQ-9 scores, 68% (20/29) had ≥6-point decreases in Generalized Anxiety Disorder-7 Scale scores, and 93% (27/29) had ≥5-point decreases in PCL-6 scores.

**Conclusions:**

PHF can be feasibly delivered within an existing intensive case management program for perinatal SUD, with early signals of mental health improvement across both intervention and control groups. Future adequately powered randomized controlled trials should investigate the effectiveness of brief behavioral interventions within perinatal SUD case management programs, optimal delivery timing, and the potential to enhance mental health care integration for high-risk perinatal populations.

## Introduction

Substance use is prevalent among pregnant and parenting individuals in the United States. Recent research indicates that 13.5% of pregnant adults currently drink alcohol, and 5.2% have engaged in binge drinking in the past 30 days [1]. Additionally, recent findings from the National Survey on Drug Use and Health show that 5.8% of pregnant women have used an illicit substance in the past month [2], and 11.3% meet criteria for a perinatal substance use disorder (SUD). Substance use in pregnancy is associated with negative outcomes, including miscarriage, teratogenicity, preterm birth, low birth weight, neonatal withdrawal syndromes, and child mental health or behavioral conditions [3].

Individuals with SUD are at greater risk of experiencing mental health conditions and vice versa [4]. Up to 50% of women with SUD have depressive symptoms [5-7] and 24%‐58% have posttraumatic stress disorder (PTSD) [8-10]. Women with SUD who have comorbid mental health conditions tend to have more difficulty engaging with SUD treatment, more chronic SUD [11], and lower rates of regaining child custody [12]. Comorbidity also increases the risk for suicidal ideation and self-harm behaviors; up to 32% of women with comorbid alcohol and drug use and mental health conditions have attempted suicide or self-harm in the past 3 months [13], and mental health conditions and substance use together are the leading cause of maternal mortality in the United States [14].

Despite the high prevalence and additive negative impacts, treatment rates for comorbid SUD and mental health conditions remain concerningly low [15]. Treating comorbid mental health conditions can help improve outcomes of SUD treatment [11], and delivering mental health treatment concurrent with SUD treatment can reduce barriers to treatment access [16]. However, there are several barriers to delivering mental health and substance use treatment concurrently [17], including a shortage of providers trained to address both conditions.

The Parent Child Assistance Program (PCAP) is a 3-year intensive case‐management program for pregnant and parenting individuals with at-risk perinatal substance use [12,18-20], first developed in Seattle in the early 1990s to reduce prenatal substance exposure. The program was built upon 3 theoretical foundations: relational theory, the transtheoretical model, and harm reduction. Relational theory underscores the importance of interpersonal relationships to clients as they grow, develop, and define themselves [21]. The PCAP model puts concepts of relational theory into practice by offering personalized, knowledgeable, and compassionate support from a single case manager (CM) who works consistently with the client for 3 years, a period long enough for the process of gradual and realistic change to occur. The transtheoretical model [22] is widely known for emphasizing that, with regard to behavior change, individuals ebb and flow in their readiness to change over time and that helping strategies will be most successful when they are tailored to the client’s readiness to change, with strong attention to building intrinsic motivation and self-efficacy. In practice, the most important way in which a PCAP CM aims to exert a positive effect on a client’s self-efficacy is by listening carefully to what is important to the client and how the client thinks about various problems, and valuing the client’s perspective. CMs then promote self-directed action by helping clients define and accomplish explicit goals toward behavioral change. PCAP intervention strategies are based on harm-reduction principles with an understanding that alcohol and drug use patterns and practices can be placed along a continuum from minimally to extremely harmful [23]. PCAP seeks to reduce the risk of harmful consequences associated with substance use. Though abstinence from substance use is not required, PCAP does view sustained abstinence as the highest possible degree of harm reduction. PCAP aims to help clients pursue long-term SUD recovery through connection to community, social, health, treatment, and recovery services. PCAP CMs are highly familiar with and well-connected to the resources available in their local communities. They meet with clients at least twice per month, in clients’ own homes whenever possible, to learn about clients’ needs and goals and to gain a working understanding of the contexts of clients’ lives. Together, PCAP CMs and clients identify goal-oriented action steps, and CMs leverage their community connections to support clients as they navigate recovery.

More than 70% of PCAP clients report having a diagnosed mental health disorder. Among these, 70% report a diagnosis of depression. More than half (58%) are involved with the child welfare system at enrollment [24]. High rates of psychiatric comorbidity in this population are especially concerning, as PCAP data indicate that those mothers with more serious psychiatric problems are less likely than other mothers to retain or regain child custody during the 3-year intervention [12]. Even with a PCAP CM, mothers’ untreated mental health problems may limit their ability to access mental health services and use other available community services to build a stable home environment for themselves and their child or children.

Task-sharing models can be used to address the treatment gap for comorbid mental health and SUD. In these models, tasks are clearly defined and distributed across larger teams. For example, lay or community health workers are trained in mental health care delivery, and specialists provide ongoing supervision, support, and consultation to frontline nonspecialist health workers [25-27]. To address the unmet mental health needs of PCAP clients, we developed a task-shared, low-intensity intervention for depression that PCAP CMs could deliver to their clients. This intervention, Promoting Healthy Families (PHF), is adapted from MOMCare, an evidence-based model using Brief Interpersonal Psychotherapy [28] found to reduce depression and anxiety in pregnant women [29] that can be delivered effectively by nonspecialists [30]. Over the course of 3 years, we tested the integration of PHF into PCAP in a pilot quasi-experimental study to assess the feasibility of PHF delivered by PCAP CMs and the preliminary effectiveness of PHF on clients’ mental health symptom severity. We hypothesized that PCAP CMs would find it feasible to deliver the PHF intervention to their clients.

## Methods

### Procedure

Participants were enrolled from April 2018 through October 2020 at 3 participating PCAP sites in Washington State (2 rural and 1 urban) [31]. PCAP eligibility criteria include age 18 years or older; pregnant or up to 24 months post partum; engaged in at-risk substance use during the most recent pregnancy; and inadequately connected to health, social, and community services. Eligibility for the study was assessed by PCAP supervisors at the time clients were enrolled in PCAP. Additional criteria to be enrolled in the study were current depressive symptomatology and either anxiety or PTSD, as evidenced by a Patient Health Questionnaire-9 (PHQ-9) score of ≥10; and a Generalized Anxiety Disorder-7 Scale (GAD-7) score of ≥10, or a PTSD (posttraumatic stress disorder) Checklist (PCL-6) score of ≥14. Clients were excluded from the study if they reported current psychotic symptoms or had permanently lost custody of the index child. Eligible clients were assigned by supervisors to either PCAP alone (control condition) or to PHF in PCAP (intervention condition) based on the availability of trained CMs to provide PHF. We continued enrollment until 60 women were enrolled. In total, 2 participants withdrew from the study before starting any study procedures, resulting in a final sample of 29 women in the PHF in the PCAP group and 29 women in the control PCAP group.

### Interventions

#### PCAP Only

Participants in the PCAP-only condition received only the PCAP intervention, which is described in detail elsewhere [12,19]. In brief, each participant was assigned to a bachelor-level CM, whose charge was to meet with the client on approximately a biweekly basis, in the client’s own home whenever possible. Together, the client and CM reviewed the client’s goals and progress toward them every 4 months. Under the guidance of a PCAP clinical supervisor, CMs provided tailored care navigation, modeling, and support, including occasional transportation to appointments in support of the client’s own goals, if needed.

#### PHF in PCAP

Participants in the PHF in the PCAP condition received both interventions. A total of 4 PCAP clinical supervisors and 8 PCAP CMs from 3 participating PCAP sites were trained by NG and MC, experts in interpersonal psychotherapy [32] and developers of the PHF intervention. Trainees received a training manual and participated in a 2-day training on the principles and methods of PHF, including strategies to help PCAP clients with symptoms of depression and anxiety, strengthen social supports, access resources to meet basic needs, and parent effectively. PHF is a culturally relevant brief behavioral intervention that teaches self-care for depression. The goals of PHF are to reduce depression symptom severity, increase and activate social support, and improve interpersonal functioning. CMs used “quick sheets” (detailed fidelity checklists based on methods established in MOMCare, a brief intervention based on Interpersonal Therapy) [33] with clients at each session to structure and guide them through the session and ensure delivery of the intervention’s essential components. We modified the quick sheets based on feedback from PCAP CMs. First, we reduced the number of problem areas, limiting it to either managing a complicated pregnancy or role transition of parenting a child, specifically addressing issues related to substance use, such as the fear of loss of child custody at birth, family and cultural myths around seeking medication treatment, and coping with loss of custody. If the problem areas of complicated grief and role dispute came up, we recommended that they be a secondary focus or that they be addressed once the clients’ depressive symptoms improved. We changed the name of the problem area role dispute to disagreements with a significant other. We focused education sessions on using 4 behavioral strategies: interpersonal behavioral activation, building effective communication skills, role coaching or role-playing, and problem solving. PHF CM used concrete tools to help build self-care skills, such as a mood chart, goal-setting worksheets, and weekly homework, focused on attaining pleasure or mastery. We facilitated access to support services, particularly around mental health counseling and medication treatment, and at times, the PHF CM would accompany the client to an initial appointment. During the intervention period, NG and MC provided virtual weekly, half-hour supervision and worked collaboratively with CMs to adapt PHF strategies to fit the context of the participants’ lives.

Table 1 illustrates the mental health intervention components of the Parent Child Assistance Program and Promoting Healthy Families models.

**Table 1.** Key mental health intervention components of the Parent Child Assistance Program and Promoting Healthy Families.

Components	PCAP^a^	PHF^b^
SUD^c^ treatment	Referral to SUD treatment in community	Referral to SUD treatment in community
Acute MH^d^ intervention	Referral for mental health or depression care in community	CM^e^ delivers PHF (weekly or every 2 weeks for 8 weeks or as needed up to 16 weeks). Assistance and encouragement with connecting with MH services and meds if desired
Maintenance MH intervention	None	Once a month for 1 year
MH outcomes tracking	PCAP intake and 3-year exit symptoms assessment using Addiction Severity Index	PHQ-9^f^ assessments at each session, GAD-7^g^ and PCL-6^h^ each session (time-permitting, if relevant), as well as tracking medications, dosage, and adherence
Supervision	Twice a month individual supervision with PCAP clinical supervisor	Weekly PHF group supervision with CM, PCAP supervisor, and PHF trainers

a PCAP: Parent Child Assistance Program.

b PHF: promoting healthy families.

c SUD: substance use disorders.

d MH: mental health.

e CM: case manager.

f PHQ–9: Patient Health Questionnaire-9.

g GAD-7: Generalized Anxiety Disorder-7 Scale.

h PCL-6: PTSD (posttraumatic stress disorder) Checklist.

### Measures

To assess feasibility, we used an 8-item anonymous online survey. Depression and anxiety symptom severity were assessed using the PHQ-9 [34] and GAD-7, respectively [35]. PTSD symptoms were assessed using the 6-item PCL-6, a brief screening tool [36] derived from items on the PTSD checklist–civilian version with evidence of good psychometric properties [37].

### Data Collection and Analysis

We collected data on perceptions of the feasibility of delivering PHF from the intervention condition PCAP staff.

To assess preliminary effectiveness, standard measures of mental health symptoms (described above) were collected at baseline and at 3 follow-up time points: 4‐5 months post baseline (mean 4.6, SD 0.4), 6‐10 months post baseline (mean 8, SD 1.4), and 12‐16 months post baseline (mean 13.8, SD 1.5). Baseline data were collected in person by a PCAP supervisor as part of PCAP intake and screening. A research assistant who was blind to the intervention assignment conducted the brief follow-up assessments by telephone. Attempts were made to collect data at each follow-up time point.

We compared means between groups, controlling for baseline scores and cumulative number of PHF sessions. In a secondary analysis, we categorized the sample into 3 groups based on how their PHQ-9, GAD-7, and PCL scores changed over the study period: improved, no change, and deteriorated. A 5-point change was used to determine a clinically significant change on the PHQ-9 [38] and PCL-6 [39], and 6 points for the GAD-7 [40]. We computed change scores from baseline through the fourth (final) timepoint. To be conservative, if a client’s scores improved at some timepoints and worsened at others over the 4 timepoints, this would be scored as “deteriorated.” Due to low base rates, we collapsed no change and deteriorated groups and conducted a Fisher exact test to assess differences between PHF in PCAP and the PCAP only. Analyses were conducted in IBM SPSS Statistics (version 19) [41].

### Ethical Considerations

We obtained approval to conduct the pilot study from the Washington State Institutional Review Board [D-030310-S, “PCAP”]. Informed consent was obtained from all participants. Study data were deidentified for analysis. Participants received a US $5 gift card for a local retailer after each completed data collection (baseline and 4, 6, and 12 months post baseline).

## Results

### Survey Results

Overall, 9 CMs and 1 PCAP supervisor responded to the anonymous survey regarding their perceptions of the feasibility of delivery. All reported that their clients seemed to have benefited from PHF (3/10 reported that their clients benefited “a lot” and 7/10 that they benefited “somewhat”). CMs indicated that PHF had affected their work “a lot positively” (60% of respondents, 6/10) “somewhat positively” (2/10 respondents), and “somewhat negatively” (2/10). When asked about their work burden changes, they noted that their PHF-related duties had increased their workload “a lot” (2/10, 20%), “somewhat” (7/10, 70%), and “not at all” (1/10, 10%).

CMs were asked about ways to reduce the workload burden that came with adding PHF. They suggested hiring new CMs that would do PHF-specific work “rather than trying to carve out with existing CMs’ caseload” or allowing for “smaller caseloads.” They also identified a need for more supervision, emotional support, and more streamlined program implementation to reduce administrative burdens associated with this added responsibility.

Survey participants suggested improving the capacity and skill levels of CMs (through more staff training in the PHF model and comorbid mental health and SUD) and increasing the amount of time available to incorporate PHF into their workload. Recommendations for improving PHF for clients included offering incentive funds and an official certificate of completion to clients. CMs also suggested that PHF enrollment should occur when clients are in active recovery, have their basic needs met (eg, housing), and when there has been enough time for relationship-building with the CM. One CM stated that:

I wonder if including PHF after the client’s life has settled down a little would be more beneficial. This might also control for situational depression/anxiety due to life instability that can be solved by meeting [more foundational] needs.

### Pilot Trial Results

**Figure 1.**
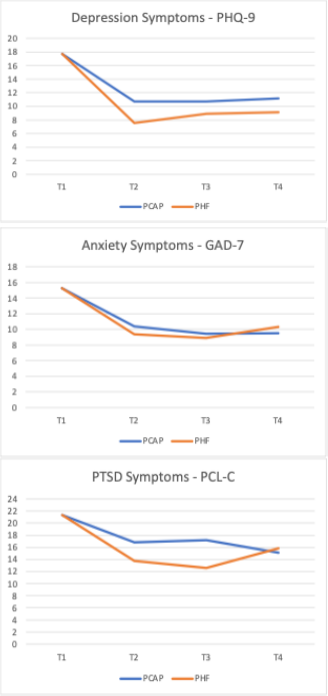
Comparison of Patient Health Questionnaire-9, Generalized Anxiety Disorder-7 Scale, and Posttraumatic Stress Disorder Checklist–Civilian mean scores between Parent Child Assistance Program and Promoting Healthy Families in Parent Child Assistance Program. GAD-7: Generalized Anxiety Disorder-7 Scale; PCAP: Parent Child Assistance Program; PCL-C: Posttraumatic Stress Disorder Checklist–Civilian; PHF: Promoting Healthy Families; PHQ-9: Patient Health Questionnaire-9.

Only 1 participant who screened eligible for the study chose not to participate, 1 participant enrolled in PHF but dropped out of PCAP before starting PHF and so became ineligible, and 1 participant dropped out of PHF before starting (but was still in PCAP). A total of 29 clients were assigned to PCAP and 29 to PHF in PCAP. When it was not possible for the woman to participate by telephone, assessments were completed online; 29% (17/58) of assessments were completed in this way. The average number of completed follow-up assessments per participant was 2.3 out of 3 (SD 1.1), and 60% (35/58) of participants completed all assessments. There were no significant differences in the number of follow-up assessments between the 2 groups. The average number of PHF sessions over 1 year, including acute and maintenance sessions, was 13.3 (SD 8.9; range 0‐34) sessions, and women received PHF services for an average of 8.5 (SD 4.7; range 0‐14) months. The mean age of participants was 28 (SD 6) years, and on average, 52% (30/58) had completed 12 years of high school and 93% (54/58) were unmarried. With regard to race and ethnicity, 58% (33/58) were non-Hispanic White, 32% (18/58) were non-Hispanic American Indian/Alaska Native/Canadian First Nations, and 10% (6/58) were non-Hispanic Black, non-Hispanic Pacific Islander, or Hispanic American Indian/Alaska Native/Canadian First Nations. Baseline mental health data are shown in Table 2. At baseline, participants in the PHF in PCAP group had marginally higher depression scores than the control group (*P*=.06). While the means were not significantly different between groups at any of the follow-up assessments, the results were in the predicted direction for the PHQ-9 from T2-T4 and for the PCL-C from T2-T3 (Figure 1). Based on biannual CM reports, participants in the PHF in the PCAP group were significantly more likely to be receiving individual mental health counseling at 24 months (n=23*; χ*²_1_=4.48; *P*=.03) as compared to those in PCAP alone.

**Table 2.** Baseline mean symptom scores on mental health measures.

Mental health measures^a^	PHF^b^ in PCAP^c^ (n=29), mean (SD)	PCAP only (n=29), mean (SD)	Significance		
			*t* test (*df*)	*P* value	
PHQ-9^d^ Depression symptom scores	17.4 (5)	15.1 (4.4)	−1.9 (56)	.06	
GAD-7^e^ Anxiety symptom scores	15.1 (4.3)	15.3 (4.1)	0.2 (56)	.85	
PCL-6^f^ PTSD^g^ symptom scores	21 (5.3)	19.8 (5.7)	−0.9 (56)	.39	

a Cut-off scores that indicate need for further assessment and intervention: PHQ-9 ≥10, GAD-7 score ≥10, and PCL-6 ≥14.

b PHF: promoting healthy families.

c PCAP: Parent Child Assistance Program.

d PHQ-9: Patient Health Questionnaire-9.

e GAD-7: Generalized Anxiety Disorder-7.

f PCL-6: PTSD (posttraumatic stress disorder) Checklist.

g PTSD: posttraumatic stress disorder.

Our final analysis was to categorize scores with respect to improved symptoms, stable symptoms, and worsening symptoms (Table 3). In our categorical analysis of depression, 85% (24/29) of the PHF in the PCAP group recorded a 5-point drop in PHQ depression score at some point from T1 during the intervention period, and none recorded a 5-point increase. In comparison, in the PCAP-only group, 71% (21/29) recorded a 5-point drop in PHQ-9 and 17% (5/29) recorded a 5-point increase. With regard to anxiety, 68% (20/29) of the PHF in the PCAP group reported a 6-point drop at some point from T1, and 7% (2/29) had a 6-point increase in scores at some point from T1. Among the PCAP-only group, 71% (21/29) reported a 6-point drop in GAD-7 scores, and 4% (1/29) of scores were worse. In our analysis of PTSD, 93% (27/29) of the PHF in the PCAP group recorded a 5-point drop in PTSD score at some point from T1, and none recorded a 5-point increase. In the PCAP-only group, 71% (21/29) recorded a 5-point drop in PTSD and 21% (6/29) recorded a 5-point increase.

**Table 3.** Clinical change in mental health through the study period by treatment group. Due to unbalanced cells and cell sizes smaller than 5, we report *P* values from the Fisher exact test.

Mental health measures and changes		PHF^a^/PCAP^b^ (n=29), n (%)	PCAP (n=29), n (%)	Test statistic	
				OR^c^ (95% CI)	*P* value
Depression				2.37 (0.60-9.40)	.31
Improvement		25 (85)	21 (71)		
No change or deterioration		4 (15)	8 (29)		
Anxiety				0.82 (0.25-2.71)	.77
Improvement		19 (67)	21 (71)		
No change or deterioration		10 (33)	8 (29)		
PTSD^d^				5.15 (0.95-27.84)	.07
Improvement		27 (93)	21 (71)		
No change or deterioration		2 (7)	8 (29)		

a PHF: promoting healthy families.

b PCAP: Parent-Child Assistance Program.

c OR: odds ratio.

d PTSD: post-traumatic stress disorder.

## Discussion

### Principal Findings

This pilot study examined the feasibility and potential impact of integrating the PHF brief behavioral intervention into PCAP for mothers with SUD. Findings suggest that CMs were able to deliver PHF within the existing PCAP framework. Although the small sample size and nonrandomized allocation limit conclusions, these initial results suggest important directions for service delivery and research in perinatal SUD care. Although no comparisons were statistically significant (likely due to insufficient power), the PTSD symptom improvement was notably higher in PHF in PCAP versus PCAP alone. The improvement in mental health symptoms across both study arms may reflect PCAP’s emphasis on connecting mothers to community services. Prior work has demonstrated that service linkage is a key predictor of improved social-emotional functioning for mothers and children [20]. Our study did not collect information on service use during the intervention, limiting our interpretation of this finding; however, it is plausible that reducing unmet needs and enhancing social support through PCAP played a role in mitigating stress and improving mental health symptoms. To the extent that connecting mothers to community services improves mental health and social-emotional functioning, activating mothers who may feel stuck due to depression or anxiety and warm handoffs to beneficial services may have a synergistic impact.

These findings carry implications for case management programs and clinicians supporting individuals with perinatal SUD. Delivering targeted brief behavioral interventions alongside SUD case management appears feasible. Embedding brief behavioral interventions into programs such as PCAP may enhance program offerings without requiring major personnel or operational changes. However, the delivery of the brief behavioral intervention may be best timed to follow stabilization of housing, safety, and basic needs. For researchers, these results suggest the need for larger randomized control trials to disentangle the effects of behavioral interventions and PCAP, with careful measurement of service use, CM contact time, and contextual disruptions such as the COVID-19 pandemic. Policymakers and funders may view the combination of SUD case management and a brief behavioral intervention as a potentially cost-effective strategy with wide-reaching benefits, including reduced mental health burden, improved parenting, and greater family preservation. Finally, and most importantly, for clients and families, timely and concurrent access to a brief behavioral intervention within the trusted case management relationship may lead to improved mental health without increasing the burden of accessing mental health treatments.

### Limitations

This study has several limitations, including the use of a nonvalidated feasibility survey, a nonrandomized design, and the absence of service use and CM contact time data. The COVID-19 pandemic disrupted core supports such as SUD treatment access, visitation with children, employment, and prenatal care, which may have reduced the measurable impact of both interventions. Staff turnover further delayed enrollment and affected continuity of care, potentially influencing participant outcomes. Future iterations of this approach would ideally take into consideration the feedback from CMs that delivering the intervention increased their workload. Despite these constraints, this pilot study offers foundational evidence that brief behavioral interventions can be delivered within PCAP and suggests potential benefits for clients with PTSD that merit further exploration. A fully powered randomized controlled trial is necessary to determine the effectiveness, optimal delivery timing, and sustainability of integrating brief behavioral interventions into SUD case management.

### Conclusions

Our findings are broadly consistent with prior research on integrating behavioral health interventions into perinatal case management. Home visiting and case management programs have demonstrated that embedding mental health components can improve maternal engagement and symptom outcomes [42,43]. The observed trends toward improvement in depression and PTSD symptoms in our study align with evidence that structured, relationship-based interventions can reduce psychological distress among mothers with SUD [44]. .Our results did not reach statistical significance, likely due to limited sample size and pandemic-related disruptions.

Addressing the challenge of comorbid SUD and mental health in the critical perinatal period by integrating interventions into existing service frameworks is a pragmatic pathway to breaking intergenerational cycles of trauma. Although preliminary, our findings highlight the promise of a low-barrier integrated approach that meets parents where they are.

## Abbreviations

CM: case manager
GAD-7: Generalized Anxiety Disorder-7 Scale
PCAP: Parent Child Assistance Program
PCL-6: PTSD (posttraumatic stress disorder) Checklist
PHF: promoting healthy families
PHQ-9: Patient Health Questionnaire-9
PTSD: posttraumatic stress disorder
SUD: substance use disorder
